# Eggs and Health Special Issue

**DOI:** 10.3390/nu8120784

**Published:** 2016-12-02

**Authors:** Maria Luz Fernandez

**Affiliations:** Department of Nutritional Sciences, University of Connecticut, Storrs, CT 06269, USA; maria-luz.fernandez@uconn.edu

In 1968, the American Heart Association recommended the consumption of no more than 300 mg/day of dietary cholesterol and emphasized that no more than 3 eggs should be eaten per week, resulting in substantial reductions in egg consumption, not just by diseased populations but also by healthy individuals, and more importantly by poor communities in undeveloped counties who were advised against consuming a highly nutritious food. These recommendations did not take into account that eggs not only contain important nutrients for overall health but also components which exert protection against chronic disease. The newly-released 2015 dietary guidelines finally took into consideration the epidemiological information and the data from clinical interventions and eliminated an upper limit for dietary cholesterol. This special issue addresses the history of the recommendations for eggs [1], the components of eggs providing beneficial effects against disease [2,3,4,5,6], the relationship between egg intake and healthy eating index [7]; the protective effects of eggs against inflammation [8] and oxidative stress [9]. Finally, the controversies surrounding egg intake and risk for diabetes are presented in a review of epidemiological data [10] and in a clinical study [11].

The history of the recommendations of dietary cholesterol and the politics behind those recommendations as well as the perception of the public and the creation of the Egg Nutrition Center in the US are thoroughly discussed alongside the lines of evidence on which the original recommendations were based [1]. The number of studies supporting the lack of evidence of an association between dietary cholesterol and risk of heart diseases and the evidence-based research associated with the elimination of dietary cholesterol from the current dietary guidelines are presented in chronological order [1].

Eggs have been recognized as functional foods due to the presence of bioactive components, which may play a role in the prevention of chronic and infectious diseases [2]. The presence of antimicrobial, antioxidant, anti-cancer and hypotensive properties are discussed in this review [2]. Phospholipids are among the bioactive components of eggs [3]. Sphingomyelin and phosphatidyl choline have been postulated to regulate cholesterol absorption and inflammation and, interestingly, the incorporation of egg phospholipids into high density lipoprotein (HDL) appears to be a major factor in the cholesterol-accepting capacity of this lipoprotein [3]. Ovotransferrin, a protein present in egg, is well known for its antibacterial properties [4]. There is evidence that ovotransferrin and its peptides possess antiviral activity, as well as antioxidant and anti-inflammatory properties [4]. In addition, egg yolk proteins including vitellogenin, lipovitellin and phosvitin have also been shown to participate in the immune defense system, capable of killing bacteria and viruses as well as promoting phagocytosis activity [5]. A study conducted in rats demonstrated that egg white protein was very useful for the recovery of iron-deficiency anemia [6]. These roles of egg proteins in protecting against bacterial infection further document the association between egg consumption and health [4,5,6].

The role of eggs on the healthy eating index (HEI) was evaluated in 139 obese post-partum Mexican American women [7]. This article details the role of eggs as a component of the diet in some of these women and how egg-eaters achieved higher HEI scores, mainly by the higher consumption of high quality protein [7]. The anti-inflammatory properties of eggs have been demonstrated in numerous studies [8]. Among the egg components with anti-inflammatory properties are: phospholipids, the carotenoids, lutein and zeaxanthin and egg proteins. The mechanisms of action of these anti-inflammatory components is discussed in detail [8]. The components of eggs that have been shown to participate in the immune defense and act as anti-inflammatory agents, have also been shown to be anti-oxidants [9]. The role of eggs as an anti-oxidant food commodity due to the presence of specific egg proteins, carotenoids and phospholipids is thoroughly discussed in this review [9].

There is controversy regarding egg consumption and patients diagnosed with diabetes [10]. While it is clear that heart disease does not increase by egg intake, some of the epidemiological data appear to find a relationship between egg intake and diabetes [10]; thus, the authors emphasize the need for more clinical interventions in patients with diabetes. A clinical study compared the effects of two distinctive breakfasts in diabetic patients in a crossover design: one egg per day or 1 cup of oatmeal per day for 5 weeks each [11]. The authors report that there were no differences in the parameters related to cholesterol or glucose metabolism between dietary interventions. However, following egg consumption, there was a reduction of liver enzymes and inflammatory markers in these patients. [11]. Thus, this study demonstrates that, for this specific population, egg intake did not increase cardiovascular disease risk but was rather protective against inflammation. It is clear that more clinical interventions are needed so that more conclusive statements can be generated regarding egg intake and risk for people with diabetes.

## References

[1] McNamara D.J. (2015). The fifty year rehabilitation of the egg. Nutrients.

[2] Miranda J.M., Anton X., Redonde-Valbuena C., Roca-Saavedra P., Rodriguez J.A., Lamas A., Franco C.M., Cepeda A. (2015). Egg and egg-derived Foods: Effects on human Health and use as functional Foods. Nutrients.

[3] Blesso C.N. (2015). Egg phospholipids and cardiovascular health. Nutrients.

[4] Giansanti F., Leboffe L., Angelucci F., Antonini G. (2015). The nutraceutical properties of ovotransferrin and its potential utilization as a functional food. Nutrients.

[5] Sun C., Zhang S. (2015). Immune-relevant and antioxidant activities of vitellogenin and yolk proteins in fish. Nutrients.

[6] Kobayashi Y., Wakasugy E., Yasui R., Kuwahata M., Kido Y. (2015). Egg protein delays recovery while ovalbumin is useful in recovery from iron deficiency anemia. Nutrients.

[7] Vega-Lopez S., Pignotti G.A.P., Tood M., Keller C. (2015). Egg intake and dietary quality among overweight and obese Mexican-American postpartum women. Nutrients.

[8] Andersen C.J. (2015). Bioactive egg components and inflammation. Nutrients.

[9] Nimalaratne C., Wu J. (2015). Hen egg as an antioxidant food commodity, a review. Nutrients.

[10] Fuller N.R., Sainsbury A., Caterson I.D., Markovik T.P. (2015). Egg consumption and human cardio-metabolic health in people with and without diabetes. Nutrients.

[11] Ballesteros M.N., Valenzuela F., Robles A.E., Artalejo E., Aguilar D., Andersen C.J., Valdez H., Fernandez M.L. (2015). One egg per day improves inflammation when compared to an oatmeal-based breakfast without increasing other cardiometabolic risk factors in diabetic patients. Nutrients.

